# ChIP on Chip: surprising results are often artifacts

**DOI:** 10.1186/1471-2164-11-414

**Published:** 2010-07-05

**Authors:** Torsten Waldminghaus, Kirsten Skarstad

**Affiliations:** 1 Department of Cell Biology, Institute for Cancer Research, The Norwegian Radium Hospital, Oslo University Hospital and University of Oslo, 0310 Oslo, Norway

## Abstract

**Background:**

The method of chromatin immunoprecipitation combined with microarrays (ChIP-Chip) is a powerful tool for genome-wide analysis of protein binding. However, a high background signal is a common phenomenon.

**Results:**

Reinvestigation of the chromatin immunoprecipitation procedure led us to discover four causes of high background: i) non-unique sequences, ii) incomplete reversion of crosslinks, iii) retention of protein in spin-columns and iv) insufficient RNase treatment. The chromatin immunoprecipitation method was modified and applied to analyze genome-wide binding of SeqA and σ^32 ^in *Escherichia coli*.

**Conclusions:**

False positive findings originating from these shortcomings of the method could explain surprising and contradictory findings in published ChIP-Chip studies. We present a modified chromatin immunoprecipitation method greatly reducing the background signal.

## Background

**Figure 1:**
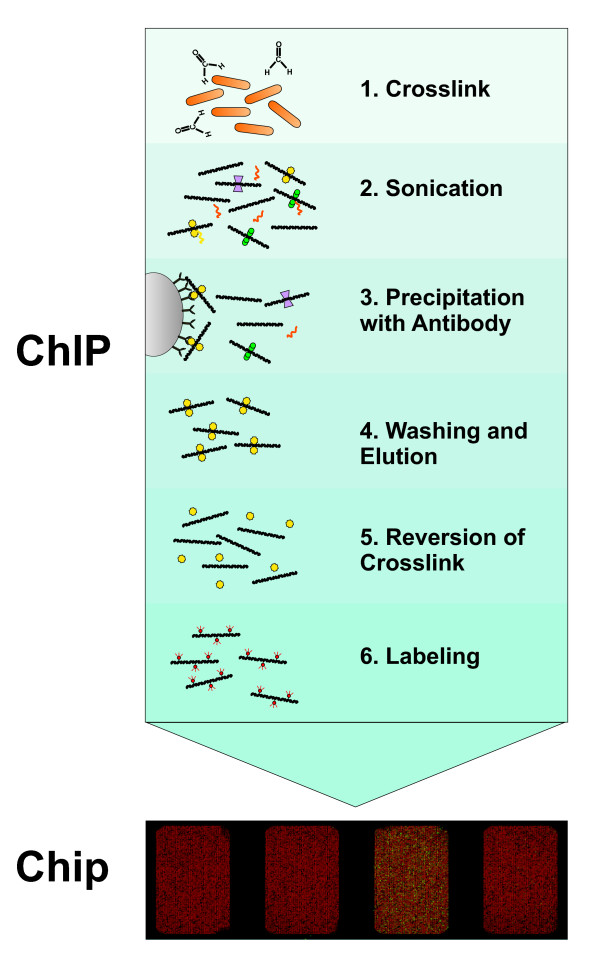
**Schematic outline of the ChIP-Chip method**. See text for detailed description.

Chromatin immunoprecipitation coupled with microarray analysis (ChIP-Chip) has become a widely used method for genome-wide localization of protein-DNA interactions [1]. Protocols have been established for different organisms with surprisingly little variation [2-5]. The first step in the ChIP-Chip procedure is to fix protein-DNA interactions in living cells by chemical crosslinking (Fig. 1). The crosslinker must be small to diffuse fast into the cells. In practice, formaldehyde is used in most ChIP-Chip experiments. After cell lysis the DNA is fragmented by sonication. This extract is then subjected to immunoprecipitation (IP) with a specific antibody against the protein of interest. DNA bound by the protein will be coprecipitated and enriched compared to DNA not bound by the respective protein. To facilitate immunoprecipitation and subsequent washing, antibodies are usually coupled to either agarose- or magnetic beads via protein A or G. After reversion of crosslinking the DNA is purified by phenol extraction or commercial PCR cleanup kits. Often, an amplification step is included after DNA purification. Two different fluorescence labels are used to label the IP DNA and a hybridization control DNA, respectively. Usually total DNA before IP (input DNA) is used as hybridization control. The two differentially labeled DNAs are hybridized to the same microarray and the difference in fluorescence intensity gives a measure of the enrichment.

We set out to investigate the genome-wide binding of the sequestration protein SeqA in *E. coli *[6]. This task can be considered especially challenging because SeqA has been shown to bind selectively to hemimethylated GATC sites [7]. Although there are about 20.000 GATCs around the *Escherichia coli *chromosome only about 2% will be hemimethylated in unsynchronized cells [8]. Such cell-to-cell variation increases the amount of cell material needed and therefore potentially the level of background signals. In fact, we found that application of a published ChIP-Chip method produced a background signal exceeding the specific signal. However, we were able to reduce the background significantly by modifying the protocol. The new protocol allowed us to uncover the genome-wide binding of SeqA and to reinvestigate σ^32 ^binding to the *E. coli *chromosome.

## Results

### High background signal in ChIP-Chip experiments

**Figure 2:**
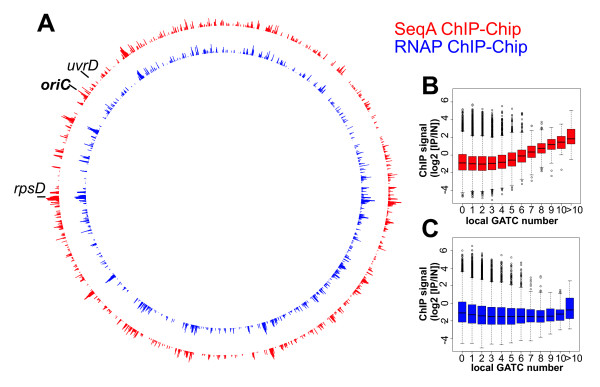
**Similar ChIP signal with SeqA and RNA polymerase antibody**. **A **Whole genome plot of RNAP and SeqA ChIP signal (log_2 _ratios of ChIP DNA/input DNA, see experimental procedures for details). **B-C **Correlation of ChIP signal with number of GATC sites. Probes were grouped according to the number of GATC sites in a region of 500 bp surrounding the probe middle position and the corresponding log_2 _ratios of ChIP DNA/input DNA are given as boxplots for SeqA (**B**) and RNA polymerase (**C**).

**Figure 3:**
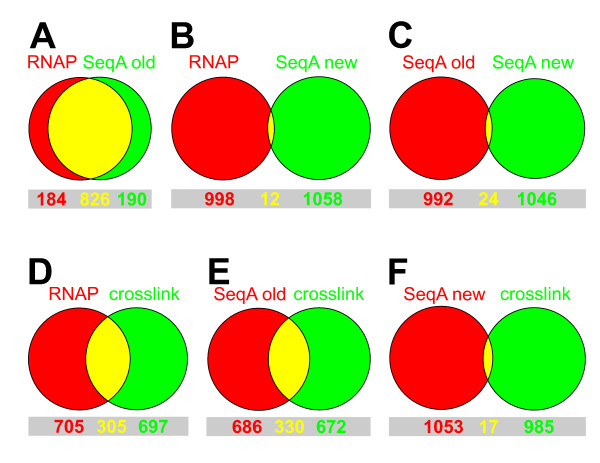
**Overlap analysis of ChIP-Chip experiments**. For each ChIP data set a cut-off was chosen to select ~1000 probes with the highest ChIP signal (or the lowest signal for the crosslinking experiment). The overlap (yellow) shows how often the signal is beyond this cut-off at similar positions in the two compared data sets (red and green). Corresponding numbers of probes are given below the Venn diagrams.

To investigate the genome-wide binding pattern of the sequestration protein SeqA in *Escherichia coli *we applied the ChIP-Chip method as described [3]. Cells were grown in LB medium, crosslinked with formaldehyde and sonicated to break down DNA to fragments of approximately 500 bps. The IP was done in parallel with antibodies against SeqA and, as a control, RNA polymerase subunit β. After reversion of crosslinking the DNA of the ChIP sample and the input DNA was differentially labeled and hybridized to a whole-genome microarray. Plotting of the ChIP signal against the genomic position revealed a great number of distinct peaks (Fig. 2). Surprisingly the binding patterns of SeqA and RNA polymerase turned out to be essentially identical (Fig. 2, compare red and blue). The overlap of the highest ChIP signals was >80% (Fig. 3A). A difference could only be seen when SeqA and RNA polymerase signals were grouped by the number of SeqA recognition sequences in the region of the corresponding probes (Fig. 2B-C). While a slight correlation between the SeqA ChIP signal and the number of GATC sites was observed at numbers of sites above 5, this was not the case for the RNA polymerase ChIP-Chip. This indicates that a specific SeqA signal is overlayed by a strong RNA polymerase-like signal in the SeqA ChIP-Chip experiment.

**Figure 4:**
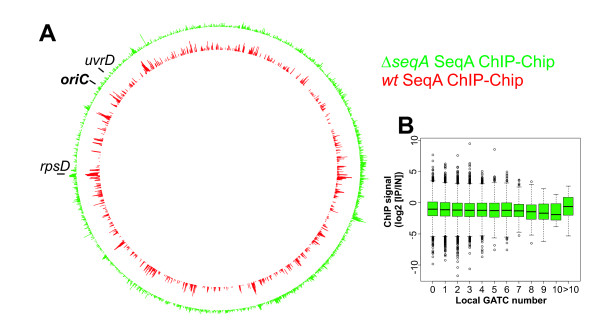
**Comparison of SeqA Chip-Chip with *wt *and Δ*seqA E. coli *reveals high background**. **A **Whole genome plot of SeqA ChIP signal for *E. coli *Δ*seqA *(green) *and E. coli wt *(red; as in Fig. 2) **B **Correlation of ChIP signal with number of GATC sites. Probes were grouped according to the number of GATC sites in a region of 500 bp surrounding the probe middle position and the corresponding ChIP signals are given as boxplots.

To estimate the degree of background signal in the SeqA ChIP-Chip we repeated the experiment using a SeqA deletion strain. All signals detected with such a set-up should be non-specific, since no SeqA protein will be present in the cell extract. The genome-wide pattern of SeqA ChIP signal in the Δ*seqA *cells showed enrichment at various regions also enriched in the *wt *cells (Fig. 4A). As expected, the former lacked the slight correlation of the ChIP signal with the local GATC number (Fig. 4B). This demonstrates that the method gave an enormous amount of background signal, exceeding the specific SeqA signal in the *wt *ChIP-Chip. Note that this background signal is not a variation of single probe intensities. It is instead the appearance of high signals in neighboring probes which is typical for a specific binding detected by ChIP-Chip.

**Figure 5:**
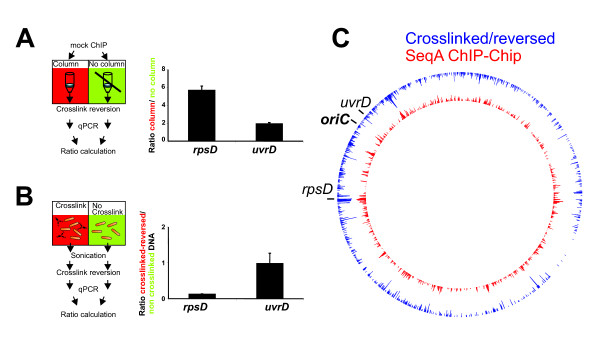
**Detection of critical steps in the ChIP-Chip protocol**. **A **Ratios of ChIP DNA purified with spin column versus column free purified DNA detected by qPCR for the indicated gene regions. **B **qPCR ratios of crosslinked-reversed versus non crosslinked DNA for indicated gene regions. **C **Crosslinked-reversed versus non crosslinked DNA as shown in B was differentially labeled and hybridized to a microarray. Log_2 _ratios are shown in blue (only values below -0.5) in comparison to the SeqA ChIP signal (from Fig. 2).

We set out to identify steps in the protocol where DNA regions giving a high background signal on the microarray behave differently compared to regions giving no background. Quantitative PCR (qPCR) was performed with the *rpsD *region which gave a high background signal on the microarray and *uvrD *which gave a low background signal (both are marked in Fig. 2). Washing turned out to be one critical step. The *rpsD *DNA was more than five-fold enriched when a spin-column was used to wash the precipitated fragments bound to agarose beads compared to when the same beads were washed without column (Fig. 5A; see materials and methods for details). Two-fold enrichment was detected for the *uvrD *region.

The background signal we observed seemed to correspond to highly transcribed regions, i.e. DNA with many RNA polymerase molecules bound (Fig. 2). Protein-rich DNA is segregated into the organic phase during phenol-chloroform extraction of crosslinked DNA [9]. However, this phenomenon should not have affected a ChIP-Chip experiment, because the crosslinking is reversed before extraction is performed. The appearance of protein-rich gene regions as background might indicate an incomplete reversion of crosslinking at these sites. To clarify this question we compared DNA that was crosslinked and reversed with DNA that was not crosslinked. (Fig. 5B; see materials and methods for details). If the reversion of the crosslinking in this protocol is complete one would expect the two signals to be the same. This was indeed the case for the *uvrD *region. However, the *rpsD *DNA was more than seven-fold reduced in the crosslinked-reversed sample compared to the non-crosslinked DNA. To analyze the effect of crosslinking and reversion on a global scale we differentially labeled the DNA and applied it to a microarray. Ratios of the crosslinked-reversed versus the non-crosslinked DNA are shown in Fig. 5C (blue signal). The results show that the same regions that gave a high background signal in the SeqA ChIP-Chip yielded a reduced signal if the DNA is crosslinked and reversed (Fig. 5C; compare blue and red signal, Fig. 3D-E).

We tested if variations of conditions influence the efficiency of crosslink reversion. Crosslinked DNA was reversed at different temperatures and with or without proteinase K (Table 1). Resulting DNA was analyzed by qPCR with *uvrD *and *rpsD *primers as above and compared to non-crosslinked DNA. As above, the *uvrD *control DNA was not changed much by crosslinking and reversion while the *rpsD *region was depleted. Notably, the level of depletion was similar for all investigated conditions. We conclude that chromosomal regions can be crosslinked to a degree which is not reversible and the respective DNA will be lost for downstream analysis.

**Table 1:** qPCR ratios of crosslinked-reversed versus non crosslinked DNA^a^

Crosslink reversion^b^	uvrD (control)	rpsD
**37°C proteinase K**	**0.36 **± 0.07	**0.03 **± 0.01
**42/65°C proteinase K**	**0.59 **± 0.25	**0.03 **± 0.01
**65°C o. n**.	**0.47 **± 0.12	**0.03 **± 0.01
**100°C 10 min**	**1.03 **± 0.44	**0.04 **± 0.02

^a^Ratios were calculated based on triplicate qPCR values for two independent cultures with the indicated standard deviation.

^b^Short description of the crosslink reversion method. See method section for details.

### Modification of the ChIP-Chip procedure allows genome-wide analysis of SeqA binding

Considering the identified weaknesses of the ChIP-Chip protocol it was possible to make appropriate modifications (see material and methods for details). The first change was the omission of spin-columns in the washing of agarose beads. Second, the input DNA was taken from the supernatant resulting from centrifugation of the immunoprecipitated chromatin beads. In addition, we included RNase digestion of immunoprecipitated DNA and excluded signals originating from microarray-probes to non-unique sequences during data analysis. The reasoning behind the latter two will be described in detail below.

**Figure 6:**
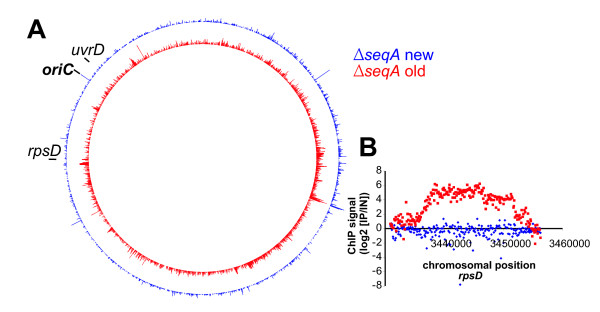
**Comparison of background signal with old and modified ChIP-Chip protocol using SeqA antiserum for immunoprecipitation of a Δ*seqA *extract**. **A **ChIP signal of the new method (blue) and the old (red). **B **ChIP signal of SeqA ChIP-Chip of Δ*seqA *for the genomic region of *rpsD*. Colors are as in A.

To test the new method we applied it to a cell extract of a *seqA *deletion strain using antiserum against SeqA (Fig. 6). As described above this should not give a specific ChIP signal and should therefore allow judgment of the level of background signal. Although some background was produced by the new method it was greatly reduced compared to the unmodified method (Fig. 6, compare blue to red). For the *rpsD *gene region the ChIP signal was reduced about 30-fold (Fig. 6B).

**Figure 7:**
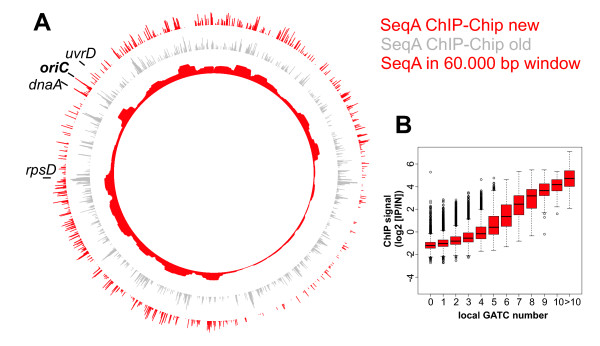
**SeqA binding to the *E. coli *chromosome**. **A **Whole genome plot of the SeqA ChIP signal with the modified method (outer red circle) in comparison to the SeqA ChIP signal resulting from old method (grey, compare fig. 2). The inner red circle is the sum of SeqA ChIP signals in windows of 60.000 bp (only positive values were included). **B **Correlation of SeqA ChIP signal with number of GATC sites per 500 bp (see legend to Fig. 2).

As a next step we used the new method to detect SeqA binding in *wt E. coli *cells. We found a distinct binding pattern with the highest peak at the origin of replication and very low SeqA binding in the terminus region of the chromosome (Fig. 7). The pattern differed greatly from that detected with the unmodified ChIP-Chip method (Fig. 7, compare red to grey, 3 B-C). Only minimal overlap with the crosslinking background was observed indicating significant reduction of background signals (Fig. 3, compare D-E with F).

To put the results in a biological context we calculated the SeqA binding signal for a 60.000 bp moving window (Fig. 7, inner ring). The reasoning behind this is that SeqA has been shown to bind specifically to hemimethylated DNA "trailing" the replication fork. We estimated the stretch of hemimethylated DNA following the replication fork to be 60.000 bp (based on a replication speed of 1000 bp/sec and an average hemimethylation time of 1 min). The result shows that SeqA binding is not evenly distributed over the chromosome. Instead there are regions with strong binding, such as the origin of replication (*oriC*) and areas with low binding, such as to the left and right of *oriC *(Fig. 7). The most extended area with low SeqA binding is about one-fourth of the chromosome around the replication terminus with distinct borders rather than smooth transitions to the neighboring high SeqA binding regions. A clear correlation was observed between the number of GATC sites in the probe region and the corresponding ChIP signal (Fig. 7B). In summary, we have shown that the revised ChIP-Chip protocol can be successfully used to gain insight into the challenging question of chromosome-wide SeqA binding in *E. coli*.

### Reinvestigation of σ^32 ^binding to the E. coli genome

Given the enormous background signal produced by the original ChIP-Chip method initially used in this study we considered it likely that published results based on this method would contain many false positives. To examine this experimentally we used our modified ChIP-Chip protocol to reinvestigate binding of the heat shock sigma factor σ^32 ^to the *E. coli *genome [10]. In the published study many novel σ^32 ^binding sites were described. Using a specific antibody we precipitated σ^32^-bound DNA from lysates of cells before and 5 min after heat shock. Of the 38 σ^32-^targets found by Wade et al. and by others in studies using alternative methods, we detected 34 (Table 2). In contrast, out of the 49 targets found exclusively in the Wade et al. ChIP-Chip study, just seven appeared in our results (Table 3). Six potential targets were detected that were not found by Wade et al., including the gene *dgsA*, also described by others (Table 4)[11]. Since application of our modified method excludes most σ^32^-targets described solely in the published ChIP-Chip study we consider it likely that these are in fact false positives (see discussion).

**Table 2:** Target detection for previously reported σ^32^-sites^a^

Genomic position^b^	Target^c^	ChIP signal^d^
12153	***dnaK***	2.9
63524	***hepA***	1.1
231081	***yafD***	1.2
415363	***sbcD***	1.3
455839	***clpP***	2.0
458088	***lon***	2.5
494367	***htpG***	2.8
517509	***ybbN***	2.2
661879	***ybeD***	2.4
692735	***ybeZ***	2.5
921161	***macB***	2.1
1027921	***yccV(hspQ)***	2.0
1120264	***yceP(dinI)***	2.1
1173268	*mfd*	-
1189625	*phoP*	-
1329105	***topA***	2.4
1338173	***yciS***	2.1
1382156	***ycjX***	2.9
1441543	*ldhA*	-
1744259	***ydhQ***	1.4
1860640	***gapA***	2.1
1910677	***htpX***	1.6
2732265	***clpB***	2.2
2748779	***grpE***	2.3
2925909	***sdaC***	1.1
3210766	***rpoD***	1.7
3325757	***ftsJ***	2.2
3437596	***yhdN***	1.3
3472778	***rpsL (yheL)***	2.3
3527175	***yrfG***	2.7
3643241	***prlC***	2.4
3865515	***ibpA***	2.4
3878830	*recF*	-
4120359	***hslV***	2.5
4366663	***fxsA***	2.4
4368634	***groE(groS)***	2.2
4397495	***miaA***	2.1
4429090	***cycA***	0.6

^a ^Targets listed were found in a previous ChIP-Chip study [10]** and **in studies using alternative methods [11,19].

^b^Chromosomal position as middle of two microarray probes. For targets not detected in this study the genomic position described by Wade et al., 2006 are given.

^c^Names of target genes with synonyms in brackets. Targets detected in this study are in bold.

^d ^Mean σ^32 ^log_2 _ratio (IP/IN) of two probes for detected targets at the indicated genomic position (see experimental procedures for details).

**Table 3:** Target detection for σ^32^-sites found only by Wade et al., 2006^a^

Genomic position^b^	Target^c^	ChIP signal^d^
22199	*ileS*	-
239254	***yafU***	1.6
516521	*ybbM*	-
705196	*glnS*	-
918375	*ybjX*	-
1063460	*yccE*	-
1213931	*ycgF*	-
1247539	*dhaM*	-
1293531	*tdk/ychG*	-
1579583	*ydeN*	-
1581861	*ydeO*	-
1584068	*ydeP*	-
1609353	*yneF*	-
1624219	*dcp*	-
1710454	*ydgR*	-
1789863	*ydiV*	-
1894985	***sdaA***	0.7
2166182	*b2084(yegR)*	-
2209265	*yehR*	-
2217821	*yehZ*	-
2236301	*mglA*	-
2288414	*narP*	-
2319337	*atoS*	-
2385514	***yfbM/yfbn***	1.0
2520600	*xapR*	-
2533473	*crr*	-
2735126	*yfiO*	-
2762841	*yfjL*	-
2764390	*yfjN(rnlA)*	-
2769847	***yfjU***	0.9
2771222	*b2641*	-
2780322	***ypjA***	1.3
2798757	*nrdH*	-
2879078	*ygcI*	-
2923838	*yqcD(queF)*	-
3117399	*yghJ*	-
3427148	*yrdA*	-
3543459	*yhgH(gntX)*	-
3725552	*yiaA*	-
3764283	*yibA*	-
3766465	*yibG*	-
4124864	*rpmE*	-
4359431	*cadC*	-
4435901	*ytfI*	-
4465355	*treR*	-
4482322	*holC*	-
4524170	***yjhI***	1.2
4539344	***fimB***	0.8
4570170	*yjiT*	-

^a ^Targets listed were found **only **in a previous ChIP-Chip study [10]

^b ^Chromosomal position as middle of two microarray probes. For targets not detected in this study the genomic position described by Wade et al., 2006 are given.

^c ^Names of target genes with synonyms in brackets. Targets detected in this study are in bold.

^d ^Mean σ^32 ^log_2 _ratio (IP/IN) of two probes for detected targets at the indicated genomic position (see experimental procedures for details).

**Table 4:** σ^32 ^target candidates not detected in Wade et al.

Genomic position^a^	Target^b^	ChIP signal
354094	*codB*	1.9
1660133	*ynfF*	0.5
1666656	***dgsA***	1.5
1798225	*rpmI*	0.5
2276466	*yejG*	0.6
4148429	*ppc*	0.8

^a ^Chromosomal position as middle of two microarray probes.

^b ^Names of target genes. Known σ^32 ^-targets are in bold according to (Nonaka et al., 2006; Zhao et al., 2005).

^c ^Mean σ^32 ^log_2 _ratio (IP/IN) of two probes for detected targets at the indicated genomic position (see experimental procedures for details).

### Limited RNase treatment is an additional source of false positives in ChIP-Chip studies

**Figure 8:**
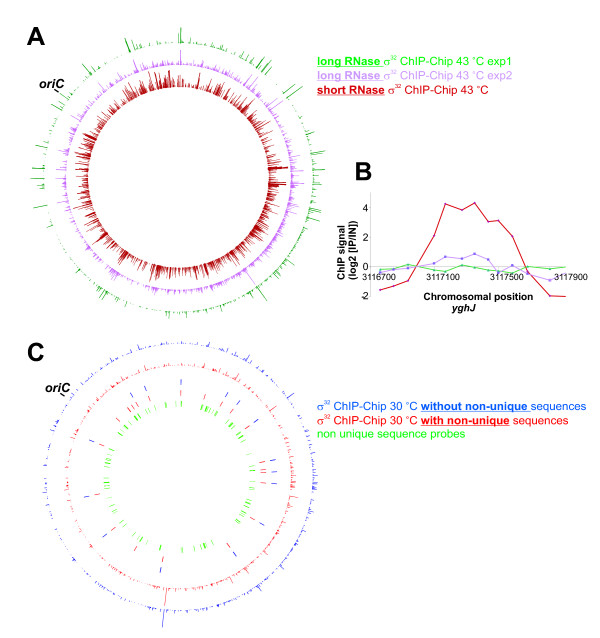
**Non-unique sequences and limited digestion of RNA cause false positives in ChIP-Chip experiments**. **A **σ^32 ^ChIP signal after long (two experiments, green and violet) and short RNase incubation (dark red). **B **σ^32 ^ChIP signal for the *yghJ *region (Colors are as in A). **C **σ^32 ^ChIP-Chip of *E. coli *grown at 30°C analyzed excluding non-unique sequences (blue) and including non-unique sequences (red). Inner three circles mark the position of peaks detected (similar coloring as for ChIP signals) and positions of non-unique sequences (green).

The σ^32 ^ChIP-Chip was used to investigate additional sources of false positive findings, such as the duration of RNase incubation of immunoprecipitated complexes. While some published ChIP-Chip protocols include an RNase digestion step others do not. We used an extended RNase incubation at 42°C for at least 90 min in our modified ChIP-Chip method. To examine the effect of limited RNA digestion we shortened the incubation to 30 min with an otherwise unchanged protocol (Fig. 8A). The shortened RNase incubation increased the unspecific background signal drastically compared to the two experiments with longer RNA digestion. Some false positive σ^32^-targets of the published ChIP-Chip study described above might originate from RNA, since the method used lacks an RNase step. Accordingly, we observed a much higher signal with shorter compared to extended RNase treatment for some of the false positive σ^32^-targets (for example *yghJ*, Fig. 8B).

### Non-unique sequences can cause false positives in ChIP-Chip analysis

One important source of false positive findings in ChIP-Chip studies is the inclusion of non-unique sequences. For the 40.000 probes on the microarray used in this study we examined the number of complementary sequences on the *E. coli *chromosome. 889 probes were found to match multiple loci on the chromosome, the numbers ranging from 2 to 11 (data not shown). Note that signals obtained with these probes and the surrounding probes were routinely excluded from all results shown above as mentioned. However, to investigate the effect of these non-unique probes we reanalyzed the σ^32^-ChIP-Chip experiment of 30°C cells described above including the non-unique sequences (Fig. 8C). Some of these probes gave an elevated ChIP signal. Consequently, six new peaks were detected by our search algorithm in addition to the 15 peaks detected before (Fig. 8C). Also the published σ^32^-study includes two target sites in non-unique sequence regions. These are the *yibA *promoter close to the *rhsA *gene and the *yrdA *promoter downstream of the ribosomal RNA gene *rrsD*. In summary, our data demonstrate the potential of non-unique sequences to cause false-positive findings in ChIP-Chip studies.

## Discussion

### Multiple sources of false positives in ChIP-Chip studies

Here we present four sources of high background signals that caused false positive target site detection in our experiments as well as in many published studies. In the following we discuss how this unspecific background might occur. The first two problems, namely the selective enrichment of some DNA fragments during spin-column washing and the variability in reversion of crosslinking, might actually be due to the same circumstance. Both affected chromosomal regions with high transcription activity, such as the ribosomal protein gene *rpsD *(Fig. 5). In such regions crosslinking of RNA polymerase, DNA and transcribed mRNA will form large complexes. Concerning the washing of immunoprecipitated DNA with spin-columns it is easy to imagine that such highly crosslinked fragments could be trapped in the column matrix. A release of these bound complexes in the elution step would explain the enrichment of protein-rich DNA through washing with spin-columns. This would be limited to the IP DNA in a ChIP experiment because usually no beads are used to purify the input DNA. The logical improvement of the protocol in this case was to wash the immunoprecipitated DNA without spin-columns. Another possibility would be to use systems which separate beads by magnetism instead of centrifugation.

In contrast, the difference in crosslinking/reversion efficiencies at genomic loci could not be reduced by leaving out the crosslinking because it is an essential part of the protocol. The incomplete reversion of crosslinking led to depletion of protein-rich chromosomal regions during DNA preparation (Fig. 5). If this depletion were similar in the IP and input DNA it would not appear as ChIP signal because the corresponding ratio would be one. However, different rates of depletion in IP and input DNA would let this ratio go up or down. If for example 60% of a crosslinked site is reversed in the IP DNA but only 30% in the input DNA this would appear as two-fold enrichment and potentially as false positive target. Thus, transcriptionally active regions of the chromosome are more likely to show a high background signal. This problem could not be solved by variation of reversion conditions (Table 1). However, as one way to better separate the real targets from such background we increased the specific signal by using the supernatant of the immuno-precipitation as input DNA. This should amplify the specific signal because it will be enriched in the immuno-precipitated DNA and at the same time reduced in the reference DNA.

A high background signal originating from non-digested RNA may also occur in ChIP-Chip experiments. This will for example be high if the Klenow fragment is used for labelling of immunoprecipitated DNA, since it can use RNA as primer to incorporate labelled nucleotides. If a linker-mediated PCR is used to amplify the immunoprecipitated DNA the amount of RNA relative to DNA will be reduced, potentially reducing the RNA-caused background. Here we show that a thorough RNase digestion is a suitable way to eliminate the RNA background, allowing a free choice of subsequent labelling and amplification techniques.

An additional origin of high background signals in microarray analysis is caused by the occurrence of non-unique sequences on the chromosomes. A systematic evaluation of labeling and microarray hybridization of predefined DNA targets revealed such genome redundancy as one major cause of false positives [12]. A probe to a non-unique sequence will bind a mix of DNA fragments originating from different chromosomal loci. The chromosomal position can influence the protein binding to the different copies of a non-unique sequence and may therefore lead to erroneous ChIP-Chip results. If for example one copy is located downstream an active promoter and the other copy not, a RNA polymerase ChIP would enrich the first locus but not the second. On the microarray this would appear as a medium enrichment at both chromosomal positions. Additional errors might occur at non-unique sequences with multiple copies and some sequence variation. In this case one probe might be complementary to for example two copies and the neighboring one to seven copies. Genes that are typically non-unique are the ribosomal and transfer RNA genes or transposons but also for example the *rhsABCD *gene family or *gadAB *in *E. coli*.

To estimate the degree of false positives caused by non-unique sequences we screened the literature for occurrence of the mentioned genes as target sites in microarray studies. Appearance of non-unique sequence false positives turned out to be quite frequent. For example, 36 out of 269 'extended protein occupancy domains' in a recent study from Vora et al. are in regions with non-unique sequences [13]. Some studies even draw major conclusions from the appearance of non-unique sequence false positives. For example, the heat shock regulator HspR was suggested to be involved in regulation of *tRNA *and *rRNA *genes in *Streptomyces coelicolor *[14], the *B. subtilis *condensin SMC was proposed to be recruited to *rRNA *and *tRNA *genes [15] and *tRNA *genes were described to be cohesin loading sites both in budding and fission yeast [16,17]. All of the mentioned gene loci are non-unique in the respective genomes. Note that in principle the described conclusions could be right; it is just that the results of microarray experiments can say nothing about it and might actually be misleading instead. Fortunately, non-unique sequences can be easily detected and corresponding probes be excluded from data sets. Even better would be elimination during array design.

Beside the causes of high background described in this study other factors have been shown to affect the background level. For example Lee and colleagues point out that ChIP-Chip experiments are highly dependent on the antibody used for the immunoprecipitation [4]. The background signal will be high if the antibody performs poorly or if it binds other proteins unspecifically. In this context the salt concentration of the IP and wash buffer is critical and can be adjusted to optimize immunoprecipitation [4]. In addition to the experimental procedure improper data processing can lead to false positive findings. How the data are analyzed will depend on different factors such as probe density and the relative number of binding sites [2]. Correct normalization regarding the dye bias in two color microarrays has been shown to be essential for ChIP-Chip experiments [18].

### How frequent are false positives in published ChIP-Chip data?

The presence of non-unique sequence false positives might indicate that a high number of false positives are the rule, rather than exception in published ChIP-Chip studies. A false positive rate about 50% was found by our reinvestigation of a published σ^32^-study [10]. The conclusion that the targets found in the published ChIP-Chip experiment but not in our study are false positives is supported by findings from others [11,19]. While almost all of the targets we detected have been found with other methods then ChIP-Chip, the only evidence for the supposed new targets by Wade et al. is their ChIP-Chip analysis [10,11,19]. It is noteworthy that this analysis was done with the protocol used in the first experiment of our study producing a high background [3]. In addition the supposed new targets lacked a typical σ^32^-recognition site [10].

Further evidence for a frequent false-positive rate in ChIP-Chip studies comes from large differences of binding site detections in parallel studies. For example, FIS was found to bind all regions on the *E. coli *genome that are bound by RNA polymerase despite the absence of consensus binding sites [20]. A later study showed very different results with data that nicely fit the distribution of FIS binding motifs [21]. In two independent studies the binding of the estrogen receptor to the human chromosome 17 of MCF-7 breast cancer cells was analyzed [22,23]. We compared the 389 binding sites described in the Gevry study to the 390 sites detected in the Carroll study and found only about 50% overlap (binding sites were considered the same when not more than 2000 bp apart, data not shown). Interestingly, others have also suggested an extended degree of false positives as explanation for contradictory results in parallel ChIP-Chip studies. Highly dissimilar binding patterns of the Mediator complex in yeast were reported [24-26]. Fan and Struhl reinvestigated the contradictory results and suggested that the differences were caused by a high degree of false positives due to the experimental set-up of Andrau and colleagues [27]. These supposed false positives are mainly located in transcriptionally active coding regions as is also the case in our study.

A high number of false positives would make systematic approaches to analyze ChIP-Chip-derived binding sites especially difficult. Indeed, a recent analysis of yeast ChIP-Chip data revealed that only 48% of detected transcription factor binding sites could be explained by direct binding and an additional 16% by indirect binding [28]. The remaining 36% of the data set could not be explained by either direct or indirect transcription factor binding and were suggested to be noise. Taken together, high false positive rates seem to be common in ChIP-Chip studies. In some cases it actually seems to be an accepted fact. For example, Partridge and colleagues removed over one third of ChIP-Chip detected NsrR target sites just because they did not fit their expectations of lying in promoter regions [29]. However, this high false-positive rate was not investigated any further.

### How to deal with the background

Beside the need for technical improvements, the high level of ChIP-Chip false positives emphasizes the great importance of suitable control experiments. Good controls are ChIP-Chip experiments with cells lacking the IP epitope (for instance Δ*seqA*; Fig. 6), mock IPs without antibody (Fig. 5A) or IPs with preimmune serum or IPs from cells growing under conditions that are expected to give no or reduced binding of the respective protein (such as 30° for the heat shock sigma factor σ^32^; Fig. 8C). A suitable control experiment has two important functions. First, it allows estimation of the experimental quality. In this study the Δ*seqA *control was the key to understanding that the ChIP-Chip method gave high background (Fig. 4 and 6). Second, a control experiment can help to detect targets in the actual experiment. We used the σ^32 ^control ChIP-Chip at 30°C to find significant targets in the corresponding data set of heat shocked cells (see materials and methods).

It has been suggested that DNA from control experiments should be used as a hybridization reference, meaning that for example the IP DNA from a *wt *strain and a deletion strain are differentially labeled and hybridized to the same array [30]. However, others point out that a control should never be used as hybridization reference [2]. We agree with the latter opinion because use of control DNA as hybridization reference would not allow assessment of the experimental quality as outlined above. For instance, bad quality DNA from experiments with limited digestion of RNA (Fig. 8A-B) might not be detected if used as hybridization reference. Taken together, appropriate control experiments should be included in every ChIP-Chip study. Submission of the raw and processed control data to the public should be self-evident but is an exception in published studies so far.

Recently, chromatin immunoprecipitation has been combined with high throughput sequencing methods (ChIP-Seq). Interestingly, an analysis of different types of control DNA resulted in a variable pattern of background distributed over the chromosomes [31,32]. The pattern of background peaks varied between input DNA, non-crosslinked DNA and mock-IP DNA and lead to the conclusion that the type of reference DNA directly influence the number of sites deemed significant when scoring ChIP-Seq data. This underlines that the described problems apply to chromatin immunoprecipitation based methods in general.

### Revised ChIP-Chip method reveals new biological insights

The revised ChIP-Chip method we developed enabled us to analyze binding of the sequestration protein SeqA to the *E. coli *chromosome. SeqA is involved in regulation of replication initiation and also proposed to play a role in chromosome organization and segregation [6]. It was found to exhibit prolonged binding to hemimethylated GATC sites at *oriC *and thereby hindering reinitiation [7,33]. Enhanced binding of SeqA at *oriC *was also found in our ChIP-Chip analysis, in fact it was the highest peak detected (Fig. 7). The second-highest peak was in the *dnaA *promoter region which has been shown to have an exceptionally long hemimethylation period [8]. While our data support SeqA binding as proposed for *oriC *and the *dnaA *promoter it contradicts published suggestions on chromosome-wide binding. Brendler and colleagues found an even distribution of potential SeqA binding sites over the chromosome [34]. Our data suggest that SeqA structures retain specific DNA tracts for varying amounts of time. Most striking is the relatively short duration of SeqA binding to the left and right of *oriC *and to the DNA at about one-quarter of the chromosome surrounding the replication terminus. The latter finding is in contrast to results from ChIP-PCR experiments with synchronized cells which suggested a prolonged SeqA binding in the terminus region [35]. Clearly, further analysis and additional experiments are needed to understand the biological meaning of the SeqA binding pattern.

## Conclusions

We describe here a revised ChIP-Chip method and show its potential to greatly reduce false positive target site detection, which seems to be a widespread problem. Although we present many examples of high false positive rates in published studies, it has to be pointed out that this will vary greatly with the exact experimental details as outlined above. Since method details such as the duration of the RNase treatment or the use of spin columns have a major impact on the background signal, it is of high importance t give an accurate description of the procedure used. The results reported here should allow critical reviewing of published ChIP-Chip studies as well as assessment and potential modification of other variants of the ChIP-Chip method and related methods.

## Methods

### Cell growth, crosslinking and preparation of cell extracts

For SeqA and RNA polymerase ChIP-Chip *E. coli *MG1655 or MG1655 *ΔseqA *(Table 5) was grown at 37°C to an OD_600 _of about 0.15 in 50 ml LB (+ 0.2% glucose) before 27 μl of formaldehyde (37%) per ml medium were added (final concentration 1%). Crosslinking was performed at slow shaking (100 rpm) at room temperature for 20 min followed by quenching with 10 ml of 2.5 M glycine (final concentration 0.5 M). For heat-shock experiments, *E. coli *MG1655 was grown in 65 ml LB medium at 30°C to an OD_600 _of about 0.3. Subsequently 30 ml of culture was transferred to a pre warmed flask at 43°C and the remainder kept at 30°C. Crosslinking and quenching was as described above except that cells were kept at 30 or 43°C for 5 min before further slow shaking at room temperature. Cells were collected by centrifugation and washed twice with cold TBS (pH7.5). After resuspension in 1 ml lysis buffer (10 mM Tris (pH 8.0), 20% sucrose, 50 mM NaCl, 10 mM EDTA, 10 mg/ml lysozyme) and incubation at 37°C for 30 min followed by addition of 4 ml IP buffer, cells were sonicated on ice with 12 times 30 sec and 30 sec breaks at an UP 400 s Ultrasonic processor (Dr. Hielscher GmbH) with 100% power. After centrifugation for 10 min at 9000 g, 800 μl aliquotes of the supernatant were stored at -20°C.

**Table 5:** Strains and oligonucleotides used in this study

Strain or oligonucleotide	Relevant characteristic(s) or sequence	Source or reference
**Strains**		
*E. coli *MG1655	F-λ rph-1 (wild type)	Guyer et al., 1981; Jensen, 1993
MG1655 *ΔseqA*	*ΔseqA10*	Torheim et al., 2000
**Oligonucleotides**		
uvrDfw	AGTTCCCGCAGGTGTTTATC	
uvrDrv	GTCAGCGTCAGTTTCTGCAT	
uvrDprobe	AGACGCCCGCCTTCATCCAG (5' FAM - 3' TAMRA)	
yahEFfw	CCATCGAGACGATCAAAGAA	
yahEFrv	CAGCATCTGGCTTTGTTGTT	
yahEFprobe	AACTCGCGTCCTTCGGCAGC (5' FAM - 3' TAMRA)	
rpsDfw	AAGTTGATGCTGGCAAGATG	
rpsDrv	TAAAGCTCGACGATCAGGTG	
rpsDprobe	TCAGAACGCTCCGGCTTACGC (5' FAM - 3' TAMRA)	

### ChIP

The ChIP protocol initially used in this study was as described in Grainger et al., 2004 except that DNA was purified with phenol/chloroform instead of a PCR clean up kit. 800 μl of sonicated cell extract (see above) was incubated with 20 μl protein A/G agarose beads (Ultralink) and 5 μl of SeqA antiserum or antibody against RNA polymerase subunit β (Neoclone) at 4°C over night. Samples were transferred to a Spin-X centrifuge column (Costar), centrifuged for 2 min at 4.000 rpm to collect the beads. The flow through was removed. Washing was done by adding 500 μl buffer to the beads on the spin column and rotation at room temperature for three minutes with subsequent collection of the beads by centrifugation as above. Washing was performed with the following buffers (IP buffer two times all others one time): IP buffer (50 mM HEPES-KOH pH 7.5, 150 mM NaCl, 1 mM EDTA, 1% Triton × 100, 0.1% Sodium deoxycholate, 0.1% SDS), IP buffer with 500 mM NaCl, wash buffer (10 mM Tris pH 8.0, 250 mM LiCl, 1 mM EDTA, 0.5% Nonidet-P40, 0.5% Sodium deoxycholate) and TE. For elution, 100 μl elution buffer (50 mM Tris (pH 7.5), 10 mM EDTA, 1% SDS) was added to the column with the beads, incubated in a 65°C water bath for 10 min and centrifuged as above. To reverse the cross link 80 μl TE and 20 μl proteinase K (20 mg/ml) were added and samples incubated for 2 h at 42 and 6 h at 65°C. DNA was purified with phenol/chloroform. To prepare the control DNA, 800 μl of sonicated cell extract was incubated at 65°C over night. 1 μl RNase A (20 mg/ml) were added and samples incubated 30 min at 65°C before extraction with phenol/chloroform. The ChIP protocol as described above resulted in the high background signal (Fig. 2 and 4).

The following modifications were applied for the other ChIP-Chip experiments. First, agarose beads were not collected on a spin column but instead at the bottom of a usual 1.5 ml eppendorf tube. The supernatant was then removed by pipetting. Second, the control DNA was taken from the supernatant resulting from centrifugation of the precipitated chromatin beads processed further as the immuno precipitated DNA after elution. Third, before addition of proteinase K, sample and control DNA were incubated with RNase A (50 μg/ml) for at least 90 min at 42°C (except in the σ^32^-analysis shown in Fig. 8A where incubation was 30 min as indicated). Incubation of 800 μl cell extract with 15 μl σ^32^- or 5 μl SeqA antiserum was for 1 h at 4°C.

### Labeling and array hybridisation

Usually DNA from six parallel immuno-precipitations (each with 800 μl extract as described) were joined and labeled with Cy3-dCTP using the Klenow fragment and random primers of the BioPrime kit from Invitrogen. An equal amount of hybridization control DNA was labeled with Cy5-dCTP. Hybridization was for about 36 h at 55°C to *E. coli *whole genome microarrays from Oxford Gene Technology. The arrays have a probe length of 60 bases and a start to start spacing of about 150 bases. ChIP-Chip analysis were made in duplicates, except the crosslink-reversion array (Fig. 5), the Δ*seqA *arrays (Fig. 6) and the shorter RNase incubation array (Fig. 8A). Please note that the array hybridized with the SeqA ChIP of the Δ*seqA *strain with the unmodified method was of poor quality but regarded sufficient for its purpose described above.

### Microarray data processing

Arrays were scanned on an Agilent SureScan High-Resolution Scanner. Spot intensities were extracted using the Feature Extraction software 10.5.1.1 from Applied Biosystems with a linear dye normalization correction method. The data were further analyzed with the statistics software R, in particular the Bioconductor package and the limma library [36,37]. The background was subtracted and data points with a value below 0 after background subtraction were removed. Ratios of g (sample) to r (control) were calculated and normalized to the array wide average. For arrays performed in duplicates the mean of the two normalized values was calculated. Probes in gene regions with non unique sequences were deleted (a list is available on request). For σ^32^-target detection data obtained from heat-shocked cells were searched for two or more neighboring probes with a log_2 _signal > 0.5 in both replicates. This resulted in 74 potential targets (34 previously described, 9 described exclusively by Wade et al., 2006, 31 not found by Wade et al.). After subtraction of log_2 _signals of the corresponding replicates from non-heat-shocked cells, 47 potential targets remained (Tables 2, 3, 4; 34 previously described, 7 described exclusively by Wade et al., 2006, 6 not found by Wade et al.). For peak detection in σ^32^-data of non-heat-shocked cells (Fig. 8C) we searched for probes with a log_2 _ratio > 1 and the one to the left and right > 0.5.

GenomeViz was used for visualization of ChiP-Chip data [38]. Data points with log_2 _ratios > 0.5 were extracted and the corresponding genome locus assigned as 1000 bp up- and down-stream of the respective probe middle. For the moving window calculation of SeqA binding the sum of positive log_2 _ratios of 60.000 bp windows were calculated with a step size of 1000 bps. Raw as well as processed data are available at the Genome Omnibus Database, accession number GSE19053. To analyze the overlap of ChIP-Chip experiments a cut-off was chosen for each data set to select ~1000 probes with the highest ChIP signal (or the lowest signal for the crosslinking experiment). The overlap is the number of probes were the signal is beyond this cut-off at similar positions in the two compared data sets.

### ChIP washing comparison

For the comparison of washing methods (Fig. 5A) 2 × 800 μl of crosslinked, sonicated MG1655 cell extract were incubated with 20 μl protein A/G agarose beads (Ultralink) without antibody for 1 h at 4°C. One of these mock IP samples was then processed with the use of spin-columns and one without as described above. Eluted DNA was purified with phenol/chloroform and analysed by quantitative PCR as described below. Note that purification of the DNA with a Qiagen PCR cleanup kit gave the same results as the phenol extraction (data not shown).

### Crosslink comparison

To compare crosslinked-reversed with non crosslinked DNA 100 ml *E. coli *MG1655 LB culture was grown at 37°C to an OD_600 _of 0.15. After collecting 50 ml as 'non crosslinked' sample, crosslinking was done as described above. Crosslinked and non crosslinked cells were washed and sonicated corresponding to the ChIP-Chip protocol above. For experiments presented in Fig. 5B and 5D, 400 μl of the sonicated extracts were mixed with 400 μl TE and incubated with 2 μl RNase A (20 mg/ml) at 42°C for 1 h. Next, 200 μl proteinase K (20 mg/ml) were added and samples incubated for 2 h at 42 and 6 h at 65°C. For experiments without proteinase K shown in table 1, 200 μl of crosslinked extract was mixed with 200 μl TE and incubated at 65°C over night or 10 min at 100°C. For the other experiments 200 μl were mixed with 160 μl TE plus 40 μl proteinase K (20 mg/ml) and incubated at 37°C over night or for 2 h at 42°C followed by 65°C for 6 h. DNA was extracted with phenol and chlorophorm and analyzed by microarray hybridization (as above) or qPCR as described below.

### Quantitative RT PCR

Reactions were carried out in triplicates of 25 μl volume each. About 10 ng DNA was used as template in 10 μl ddH_2_O and added to a mix of 12.5 TaqMan Gene Expression mix (Applied Biosystems) and 2.5 μl primer mix (9 μM each forward and reverse primer and 2.5 μM probe) in 96 well PCR plates. For a primer list see Table 5. Reactions were carried out with a 7500 Real Time PCR System (Applied Biosystems). The system software was used to calculate Ct values which were transformed to relative values of template DNA. qPCR values for the *yahEF *gene region were used for normalization.

## Abbreviations

ChIP-Chip: chromatin immunoprecipitation combined with microarrays; ChIP-Seq: chromatin immunoprecipitation combined with next generation sequencing; IP: immunoprecipitation.

## Authors' contributions

TW designed and carried out the experiments, analyzed the data and drafted the manuscript. KS participated in design of the study, interpretation of data and in writing of the manuscript. Both authors read and approved the final manuscript.
